# Supplementary material for: Multiplex Analysis to Unravel the Mode of Antifungal Activity of the Plant Defensin HsAFP1 in Single Yeast Cells
Source: Int J Mol Sci. 2022 Jan 28;23(3):1515. doi: 10.3390/ijms23031515 (PMC8836000; doi:10.3390/ijms23031515)
Supplement: Supplementary file 1 [file ijms-23-01515-s001.zip › ijms-1548240-supplementary.pdf]

Supporting information

Multiplex analysis to unravel the mode of antifungal activity of the plant defensin HsAFP1 in single yeast cells

Caroline Struyfs[1], Jolien Breukers[2], Dragana Spasic[2], Jeroen Lammertyn[2], Bruno P.A. Cammue[1] and Karin Thevissen[1,*]

[1] Centre of Microbial and Plant Genetics, KU Leuven, Kasteelpark Arenberg 20, 3001 Leuven, Belgium
[2] Department of Biosystems, Biosensors Group, KU Leuven, Willem de Croylaan 42, 3001 Leuven, Belgium
* Correspondence: karin.thevissen@kuleuven.be; Tel.: +32 16 32 96 88

Supplementary video S1. Video of exemplary time series of yeast cells treated with 50 μg/mL HsAFP1 stained with DHE to evaluate the induction of reactive oxygen species.

Supplementary video S2. Video of exemplary time series of yeast cells treated with 50 μg/mL HsAFP1 stained with SYTOX to evaluate the induction of membrane permeabilization.
